# Copy number variations (CNVs) identified in Korean individuals

**DOI:** 10.1186/1471-2164-9-492

**Published:** 2008-10-18

**Authors:** Tae-Wook Kang, Yeo-Jin Jeon, Eunsu Jang, Hee-Jin Kim, Jeong-Hwan Kim, Jong-Lyul Park, Siwoo Lee, Yong Sung Kim, Jong Yeol Kim, Seon-Young Kim

**Affiliations:** 1 Medical Genomics Research Center, KRIBB, 52 Eoeun-dong, Yuseong-gu, Daejeon 305-806, Republic of Korea; 2 Department of Medical Research, KIOM, 483 Expo-ro, Yuseong-gu, Daejeon 305-811, Republic of Korea

## Abstract

**Background:**

Copy number variations (CNVs) are deletions, insertions, duplications, and more complex variations ranging from 1 kb to sub-microscopic sizes. Recent advances in array technologies have enabled researchers to identify a number of CNVs from normal individuals. However, the identification of new CNVs has not yet reached saturation, and more CNVs from diverse populations remain to be discovered.

**Results:**

We identified 65 copy number variation regions (CNVRs) in 116 normal Korean individuals by analyzing Affymetrix 250 K Nsp whole-genome SNP data. Ten of these CNVRs were novel and not present in the Database of Genomic Variants (DGV). To increase the specificity of CNV detection, three algorithms, CNAG, dChip and GEMCA, were applied to the data set, and only those regions recognized at least by two algorithms were identified as CNVs. Most CNVRs identified in the Korean population were rare (<1%), occurring just once among the 116 individuals. When CNVs from the Korean population were compared with CNVs from the three HapMap ethnic groups, African, European, and Asian; our Korean population showed the highest degree of overlap with the Asian population, as expected. However, the overlap was less than 40%, implying that more CNVs remain to be discovered from the Asian population as well as from other populations. Genes in the novel CNVRs from the Korean population were enriched for genes involved in regulation and development processes.

**Conclusion:**

CNVs are recently-recognized structural variations among individuals, and more CNVs need to be identified from diverse populations. Until now, CNVs from Asian populations have been studied less than those from European or American populations. In this regard, our study of CNVs from the Korean population will contribute to the full cataloguing of structural variation among diverse human populations.

## Background

Understanding variations in the human genome is the key to unraveling the phenotypic diversity among individuals and understanding various human diseases. Genomic variations exist at various levels, from differences in single nucleotides to microscopic chromosome-level variation [1]. Copy number variations (CNVs), a new type of genomic variation that has recently received considerable attention, are deletions, insertions, duplications, and more complex variations ranging from 1 kb to submicroscopic sizes [1-4]. Recent advances in array technologies such as BAC arrays, oligonucleotide array CGHs, and whole-genome SNP arrays, have finally enabled researchers to identify this new type of variation, which had gone unnoticed for a long time [5].

Since Sebat et al. [6] and Iafrate et al. [7] first reported large-scale CNVs among normal human individuals in 2004, and since then, many researchers have identified novel CNVs using diverse technical and computational approaches [8-17]. These reported CNVs are collected and maintained in a curated database, the database of genomic variants , which contains more than 15,000 CNVs obtained from 48 publications as of April, 2008. However, the discovery of new CNVs has not yet been saturated, and many challenges remain for the standardization of CNV discovery [18,19]. The global map of CNVs from the 270 normal individuals in the HapMap collection is an important advance in the field, yet genomes from more individuals from diverse populations should be studied to achieve a full cataloging of human CNVs [11].

Whole-genome SNP arrays such as Affymetrix 500 K or Illumina 300 K arrays, which are widely used for whole-genome association studies, are also useful for CNV discovery since the intensity of the probes can be exploited to detect CNV gains and losses [20-23]. A few recent studies successfully utilized whole-genome SNP data from control populations in North American and European countries for the detection of novel CNVs [19,22,24,25]. Here, we report the identification of 10 novel CNVs from 116 normal Korean individuals by analyzing Affymetrix 250 Nsp SNP array data. Our work will be valuable in expanding our knowledge of CNVs across diverse populations and ethnicities.

## Results and discussion

### CNVRs from the Korean population

Commonly used algorithms for CNV detection from SNP arrays can produce widely different results from the same data because they differ both in the way reference samples are prepared and in their calling criteria [19,26]. A stringent criterion to select only regions identified by more than two different algorithms is currently recommended to increase confidence in the identified CNVs [19]. In this work, we applied three algorithms, CNAG [21], dChip [27] and GEMCA [20], to our data set of 116 normal Korean individuals genotyped using Affymetrix 250 K Nsp arrays. We identified a total of 65 CNVRs, among which 10 CNVRs (15.4%) were novel and not present in the Database of Genomic Variants. Many novel CNVs were likely missed by our approach, but we chose to be conservative in our selection of CNVs to reduce false positives. More than 15.4% of the identified CNVs in the Korean population would be novel if we consider a recent study, which showed that most CNV loci are actually smaller than currently recorded in the Database of Genomic Variants [28].

**Figure 1:**
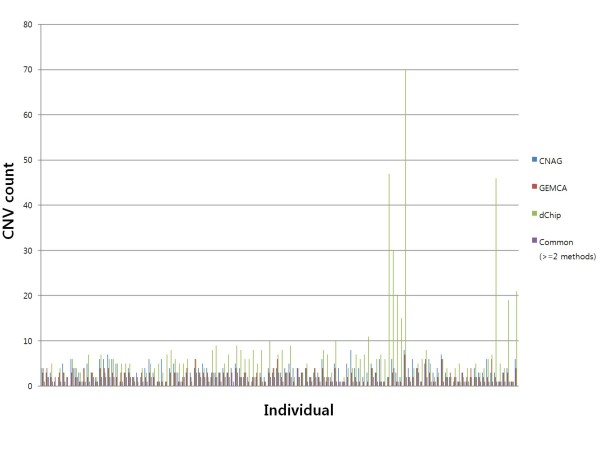
**Distribution of CNV counts identified using CNAG, dChip and GEMCA algorithms.** Distribution of CNV counts in each individual. The Y-axis represents the CNV count and the X-axis represents each individual.

**Figure 2:**
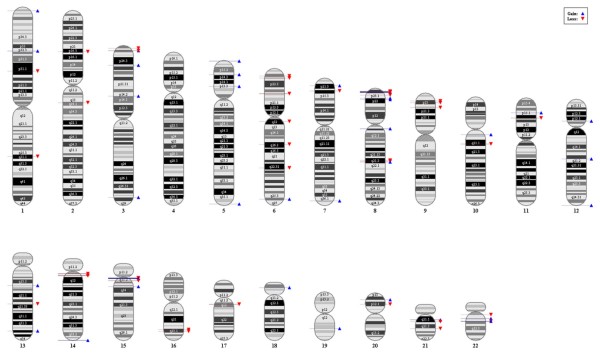
**Distribution and frequencies of CNVs identified in Korean population in the human genome.** The blue triangle indicates gains and the red inverted triangle indicates losses, respectively.

As expected, there were significant differences in the numbers and positions of CNVs identified by the three methods (Figure 1). In most cases, the dChip algorithm identified more CNVs than CNAG and GEMCA. Average 6.7, 3.5 and 2.6 CNVs per individual were found by dChip, CNAG and GEMCA, respectively (Additional file 1). In total, 772, 403 and 302 CNVs were found by the dChip, CNAG and GEMCA algorithms. Detailed information for each identified CNV is shown in Additional file 2. A total of 141 CNVs was identified by our criterion of selecting CNVs represented by more than two algorithms. When we compared size distribution between 84 duplicated and 57 deleted CNVs (Additional file 2), we found that duplicated regions had a tendency to be longer than deleted regions (p < 0.0009664, *t*-test). When we plotted each CNV in the genome, we found that most CNVs were located near the band of each chromosome (Figure 2). Finally, we defined 65 CNVRs from the 141 CNVs by merging overlapping CNVs from different individuals (Additional file 3 and 4).

### Size and occurrence of CNVs in the Korean population

The sizes of the 141 CNVs ranged from several kb to several megabases (Table 1). The smallest CNV was 15,723 bp, and the largest 2,262,135 bp. Many CNVs were in the range of 10 kb to 300 kb. We also compared the size distributions of the CNVs identified by each method. The smallest, median, and largest CNVs were 998, 153,137 and 2,264,086 bp for the GEMCA algorithm, 1,184, 267,962 and 23,992,731 bp for the CNAG algorithm and 641, 67,372 and 5,035,303 bp for the dChip. In general, CNVs identified by the dChip algorithm had larger range than those identified by the GEMCA and CNAG algorithms.

**Table 1:** Distribution of CNV sizes identified in the Korean population

Size (bp)	Novel CNV	Known CNV	Sum
10 K–100 K	3	33	36
100 K–200 K	3	58	61
200 K–300 K	3	8	11
300 K–400 K	0	8	8
400 K–500 K	1	4	5
500 K–1 M	0	7	7
1 M–10 M	0	13	13
Total	10	131	141

Most CNVs (75%) from the Korean population were rare (<1%), occurring just once among the 116 individuals (Table 2). However, a few previously reported CNVs occurred in a significant proportion of the Korean population. For instance, one CNV on chromosome 14 was present in 31 individuals. Generally, there were more CNV gains than losses, and 5 (31%) of the 16 CNVRs had mixed gains and losses among different individuals. Among all autosomal chromosomes, CNVs were detected most frequently on chromosomes 14, 15 and 8.

**Table 2:** Occurrence of CNVs among the Korean population

Occurrence	Novel CNV	Known CNV	Sum
1	10	39	49
2	0	7	7
3	0	2	2
4	0	2	2
5	0	1	1
6	0	0	0
7	0	2	2
8	0	0	0
9	0	0	0
10	0	0	0
11–20	0	1	1
21–30	0	0	0
31–40	0	1	1

Total	10	55	65

### Comparison by ethnicity

Affymetrix 500 K CEL files from the 270 HapMap individuals were obtained from the Affymetrix web site and analyzed with the CNAT algorithm to identify CNVs at an individual level. Also, individual-level CNV data from the 269 HapMap samples obtained by the array CGH method were downloaded from the copy number variation project at the Welcome Trust Sanger institute web site . The 270 individuals were divided into three ethnic groups – Asian (JPT + CHB), European (CEU), and African (YRI), and the overlap of CNVs between the Korean population and each of the three ethnic groups was investigated (Table 3). Overall, there was a 23–40% overlap in counts and a 23–79% overlap in actual nucleotides in CNVs between the Korean population and the three ethnic groups. The Korean population showed the highest degree of CNV overlap with the Asian population, as expected, but the overlap was less than 40%, implying that many more CNVs remain to be identified from the Asian population beyond those identified in the 90 Asian HapMap individuals.

**Table 3:** Overlap between CNVs from the Korean population and CNVs from the 270 HapMap individuals

Ethnicity	HAP* CNV count	HAP CNVR count	HAP CNV size (bp)	KOR# unique count	KOR-HAP overlap count	KOR-HAP overlap size (bp)	KOR-HAP overlap count percent	KOR-HAP overlap size percent
A. Affymetrix 500 K								
CHB+JPT	1,957	593	394,995,510	39	26	7,326,741	40.00%	45.60%
CEU	1,700	305	231,661,002	50	15	3,634,120	23.08%	22.62%
YRI	1,627	318	236,857,444	45	20	4,576,087	30.77%	28.48%
B. WGTP								
CHB+JPT	5,753	990	361,340,796	42	23	12,679,720	35.38%	78.91%
CEU	5,597	970	358,358,246	50	15	6,486,626	23.08%	40.37%
YRI	7,280	469	170,541,572	46	19	4,482,724	29.23%	27.90%

*HAP: HapMap individuals.

#KOR: Korean population

### Novel CNVRs from the Korean population

Among the 10 novel CNVRs identified from the Korean population, 3 CNVRs contained a total of 5 genes (Additional file 5). The total length of the novel CNVRs was 1,788,129 bp, or 0.06% of the human genome. The total length of the 55 known CNVRs is 14,280,140 bp (0.48% of the human genome). Twenty-four of these CNVRs contained 52 genes.

**Figure 3:**
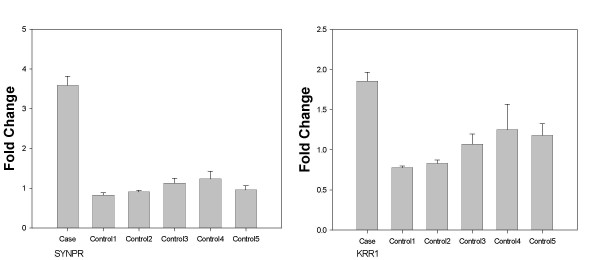
**Validation of two novel CNVRs by Q-PCR.** (A) SYNPR, (B) KRR1. For each gene, one case (with a CNVR) and five normal controls were compared by Q-PCR. The mean of five control samples was arbitrarily set to 1 and each sample was compared to the mean value. Each bar and error bar represents a mean and standard deviation of relative expression values from triplicate experiments.

Among the three novel CNVRs, we validated two CNVRs by Q-PCR (Figure 3). One case sample, which had a gain of two copies in a novel CNVR encompassing SYNPR gene, showed a 3.59-fold increase in DNA copy number in comparison to five samples with normal copy number (Figure 3A). The other validated region was a CNVR containing KRR1 gene, In this case, the case sample, which had a gain of one copy, showed a 1.86-fold increase in DNA copy number in comparison to five samples with normal copy number (Figure 3B).

We analyzed the functional enrichment of genes contained in the CNVRs from the Korean population using the GOstat tool (Table 4 and 5) [29]. The novel CNVRs were enriched with genes involved in regulation and development processes (Table 4). Genes in the previously known CNVRs were mainly related to processes such as cell adhesion, multicellular, development, and regulation of gene expression (Table 5). Our results are in agreement with Nguyen et al.'s work, which showed the over-representation of secreted, cell adhesion, and immunity-related proteins in CNV-associated genes [30].

**Table 4:** Functional annotation of novel CNVs from the Korean population

**GO id**	**Group count**	**Total count**	**P-value**	**GO terms**
GO:0045995	1	8	0.033	regulation of embryonic development
GO:0050679	1	18	0.0333	positive regulation of epithelial cell proliferation
GO:0050678	1	30	0.0333	regulation of epithelial cell proliferation
GO:0050673	1	33	0.0333	epithelial cell proliferation
GO:0030334	1	60	0.0333	regulation of cell migration
GO:0030155	1	66	0.0333	regulation of cell adhesion
GO:0051270	1	69	0.0333	regulation of cell motility
GO:0040012	1	72	0.0333	regulation of locomotion
GO:0040011	1	73	0.0333	locomotion
GO:0016477	1	233	0.0738	cell migration
GO:0008284	1	233	0.0738	positive regulation of cell proliferation
GO:0009790	1	235	0.0738	embryonic development
GO:0050793	1	236	0.0738	regulation of developmental process

**Table 5:** Functional annotation of known CNVs from the Korean population

**GO id**	**Group count**	**Total count**	**P-value**	**GO terms**
GO:0007565	5	81	7.62E-06	female pregnancy
GO:0007608	7	540	0.000303	sensory perception of smell
GO:0007606	7	584	0.000336	sensory perception of chemical stimulus
GO:0022414	5	305	0.00128	reproductive process
GO:0051704	5	333	0.00156	multi-organism process
GO:0007600	7	950	0.00365	sensory perception
GO:0007165	15	5142	0.0117	signal transduction
GO:0007166	10	2433	0.0117	cell surface receptor linked signal transduction
GO:0050877	7	1269	0.0134	neurological system process
GO:0007186	8	1677	0.0134	G-protein coupled receptor protein signaling pathway
GO:0007154	15	5560	0.0237	cell communication
GO:0006433	1	2	0.0264	prolyl-tRNA aminoacylation
GO:0003008	7	1539	0.0286	system process
GO:0017000	1	3	0.0339	antibiotic biosynthetic process
GO:0016999	1	4	0.0421	antibiotic metabolic process
GO:0009649	1	5	0.0493	entrainment of circadian clock
GO:0007185	1	6	0.0498	transmembrane receptor protein tyrosine phosphatase signaling pathway
GO:0006426	1	6	0.0498	glycyl-tRNA aminoacylation
GO:0017144	1	6	0.0498	drug metabolic process

The fact that 15% (10/65) of CNVs in the Korean population were novel implies that current CNV discovery has not yet plateaued, and that the genomes of more individuals should be examined to fully understand CNVs in the general population. Until recently, CNV studies have mainly focused on populations in North America and Europe [19,25]. More individuals from other continents, such as Asia, Africa, and South America, need to be studied to enrich our understanding of the diversity of CNVs in the human population. We stress that the Korean population had less than a 40% overlap in CNVRs with the 90 Asian HapMap individuals, which suggests that more individuals should be studied to fully represent the pattern of CNVs among East Asian populations. In this regard, our work on 116 Korean individuals will be a useful resource for better understanding the diverse variation in the human genome.

## Conclusion

Recent studies have shown that CNVs are as important as single nucleotide polymorphisms (SNPs) or microscopic variations. Many studies have reported the identification of novel CNVs, but more CNVs from diverse populations should be identified until we have a full catalogue of the structural variations among human populations. Until now, the CNVs of Asian populations have not been as thoroughly studied as those of European or American populations, and in this regard our study of CNVs from the Korean population will contribute to the full cataloguing of structural variations among diverse human populations.

## Methods

### DNA samples

Blood specimens were obtained from normal, healthy subjects who visited the Korean Institute of Oriental Medicine (KIOM) and collaborative hospitals. The internal review board at KIOM approved study protocols and informed consent was obtained from all enrolled study subjects. Genomic DNA was extracted from blood samples using the QIAamp DNA Blood Maxi Kit (Qiagen, Valencia, CA) according to the manufacturer's instruction. DNA concentration and purity were determined using the NanoDrop DN-1000 spectrophotometer (NanoDrop Technologies, Rockland, DE).

### Affymetrix GeneChip Nsp 250 K Mapping Array data

The 250 K Nsp mapping assay was performed according to the manufacturer's protocol. Briefly, DNA (250 ng) was digested with NspI (NEB, MA) and then ligated with an NspI linker supplied by Affymetrix. The ligated DNA was diluted four-fold and PCR-amplified using a PCR primer complementary to the linker DNA. The PCR products were purified using a DNA Amplification Clean-Up Kit (Clontech, CA) and 90 μg of the PCR products were fragmented by DNase I treatment. The fragmented DNA was labelled using 0.86 mM GeneChip DNA labelling reagents (Affymetrix) and 1.5 U/μl terminal deoxy-nucleotidyl transferase (TdT) for 4 hr at 37°C, while the remaining 4.5 μl was examined on 4% TBE agarose gel to confirm that average DNA fragment size was < 180 bp. Hybridization and subsequent steps were performed according to the manufacturer's instructions. Hybridization experiments that passed the genotyping call rate over 93% by the dynamic model algorithm were used in the subsequent analysis to reduce false positive predictions arising from low quality genotyping data.

### Copy number analysis using CNAG, dChip and GEMCA

Three algorithms, CNAG (version 2.0), GEMCA (available at ) and dChip, were used to infer copy numbers from 250 K Nsp SNP array data.

A reference data set of 48 normal individuals (obtained from the Affymetrix website) was used in the non-paired reference analysis with default parameters and CNVs inferred as more than two consecutive SNPs in CNAG analysis. In the GEMCA analysis, a reference data set of 10 normal individuals was used in the non-paired reference analysis and the default parameters were used. The boundary of CNVs was determined using 90% density borders [20]. Analysis with dChip was normalized at the probe intensity level with an invariant set normalization method [27]. A signal value was calculated for each SNP using an average model method (PM/MM difference). From the raw copy numbers, the inferred copy number was estimated by using HMM (Hidden Markov model) and 10% of sample trimmed options and CNVs were inferred as more than two consecutive SNPs. Finally, for each individual, CNVs were defined as a region identified by more than two algorithms (overlap rate >= 50%, length >= 1000 bp). This strategy is likely to increase a confidence in the detected CNVs although many novel CNVs may be missed [19]. Considering the current lack of standards in CNV discovery methods, we think that a more stringent approach like ours is appropriate. NCBI genome build 36 (hg18) was used to map each CNV to its genomic position.

### Comparison of Korean CNVs with those of 270 HapMap individuals

CEL files for the 270 HapMap individuals were downloaded from the Affymetrix web site. For copy number analysis of the 270 HapMap samples, the same reference set of 48 samples was used in the CNAT analysis. CNV data for each of the 269 HapMap individuals investigated using the whole genome TilePath (WGTP) array was downloaded from the CNV Project web site at the Welcome Trust Sanger Institute [11].

### Determination of novel CNVRs and functional annotation analysis

CNVs identified in our Korean population were compared with 11,966 CNVs in the Database of Genomic Variants (downloaded as of Feb. 2008). The GOstat web service was used for gene ontology (GO) term analysis to study the enrichment of GO terms in the known and novel CNVs [29]. This analysis was performed with the default option for biological processes and the GO term candidates were ordered by p-value.

### Quantitative-PCR (Q-PCR) for CNVs validation

Two selected novel CNVs were validated by Q-PCR. Q-PCR was done in 20 μl with the following components: 7.0 μl of molecular biology grade water (Hyclone, US), 10 μl of 2 × SYBR Green Premix EX Taq solution, 0.5 μl of forward and reverse primers (10 pmol/μl each) and 2 μl template DNA (1 ng/ml). Primer sequences were 5'-AGCCAGCTATCAGGTGAGGA-3' (SYNPR-forward), 5'-ACTTGTCTAAGCCCCTGCAA-3' (SYNPR-reverse), 5'-GAGTGGGCTTTGTGGTGAAT-3' (KRR1-forward) and 5'-TGTGCTGGGCATATTAGTGG-3' (KRR1-reverse). Q-PCR was conducted using CFX96 (Bio-Rad Laboratories, US) with the following cycling condition: initial denaturation at 95°C for 3 min followed by 45 cycles of 95°C for 10 s, 60°C for 20 s and and 72°C for 20 s. The relative quantification in each sample was determined.

## Authors' contributions

SL and HYP collected blood samples and prepared DNA. HJK and JHK performed genotyping experiments. JHK and JYP performed RT-PCR experiments. TWK, YJJ, and SYK performed bioinformatics analyses. TWK, YJJ, SYK, JYK and YSK wrote the manuscript. All authors read and approved the manuscript.

## Supplementary Material

Additional file 1 **Comparison of CNV counts between CNAG, dChip and GEMCA algorithms in 116 Korean individuals.** Click here for file

Additional file 2 **Detailed information on individual CNVs identified using the CNAG, dChip and GEMCA algorithms.** NCBI genome build 36 was used for chromosomal positions. Click here for file

Additional file 3 **Identification of intersected CNVs between the CNAG, dChip and GEMCA algorithms.** Click here for file

Additional file 4 **Detailed information on CNVRs identified from the Korean population.** Click here for file

Additional file 5 **A list of genes contained in CNVRs identified from the Korean population.** Click here for file
